# PCA-based sub-surface structure and defect analysis for germanium-on-nothing using nanoscale surface topography

**DOI:** 10.1038/s41598-022-11185-w

**Published:** 2022-05-03

**Authors:** Jaewoo Jeong, Taeyeong Kim, Bong Jae Lee, Jungchul Lee

**Affiliations:** 1 Department of Mechanical Engineering, Korea Advanced Institute of Science and Technology, Daejeon, 34141 South Korea; 2 Center for Extreme Thermal Physics and Manufacturing, Korea Advanced Institute of Science and Technology, Daejeon, 34141 South Korea

**Keywords:** Nanoscience and technology, Techniques and instrumentation, Characterization and analytical techniques

## Abstract

Empty space in germanium (ESG) or germanium-on-nothing (GON) are unique self-assembled germanium structures with multiscale cavities of various morphologies. Due to their simple fabrication process and high-quality crystallinity after self-assembly, they can be applied in various fields including micro-/nanoelectronics, optoelectronics, and precision sensors, to name a few. In contrast to their simple fabrication, inspection is intrinsically difficult due to buried structures. Today, ultrasonic atomic force microscopy and interferometry are some prevalent non-destructive 3-D imaging methods that are used to inspect the underlying ESG structures. However, these non-destructive characterization methods suffer from low throughput due to slow measurement speed and limited measurable thickness. To overcome these limitations, this work proposes a new methodology to construct a principal-component-analysis based database that correlates surface images with empirically determined sub-surface structures. Then, from this database, the morphology of buried sub-surface structure is determined only using surface topography. Since the acquisition rate of a single nanoscale surface micrograph is up to a few orders faster than a thorough 3-D sub-surface analysis, the proposed methodology benefits from improved throughput compared to current inspection methods. Also, an empirical destructive test essentially resolves the measurable thickness limitation. We also demonstrate the practicality of the proposed methodology by applying it to GON devices to selectively detect and quantitatively analyze surface defects. Compared to state-of-the-art deep learning-based defect detection schemes, our method is much effortlessly finetunable for specific applications. In terms of sub-surface analysis, this work proposes a fast, robust, and high-resolution methodology which could potentially replace the conventional exhaustive sub-surface inspection schemes.

## Introduction

Silicon-on-nothing (SON)^1–3^ and germanium-on-nothing (GON)^4,5^ are established as a promising fabrication methodology, with their main advantage of unmatched process simplicity for fabricating micro and nanoscale cavities^6,7^. Depending on the initial DRIE hole patterns, the annealed cavities will form a shape of either a sphere, a circular pipe, or a plate^8^. The novel fabrication process enables simple manufacturing of multiscale (micro to nano) cavities without hermetical sealing process. Due to such unique advantages, SON and GON structures are adopted in various applications^9–15^. These applications are widely sorted into two categories: devices which exploit the cavity itself and others which value its peripherals. The individual cavities formed in the initial annealing stage are utilized as photonic crystals^9^, the merged cavities are utilized as absolute pressure sensors by deflection of the membrane^10–12^ and flow channels for microcapillaries and resonators^13–15^. Other applications that value the cavity’s peripherals remove and handle the self-assembled membrane in a separate manner to fabricate solar cells^4,16,17^ and semitransparent silicon films^18^. Appropriate employment of these devices from SON and GON structures require a precise degree of cavity saturation, with specific morphologies of both surface and sub-surface structures. In that sense, a robust inspection scheme is essential for practical use of SON and GON structures.

S ince the cavity is buried under the covering layer, an accurate yet non-destructive inspection methodology is in demand to scrutinize the topography and thickness of the self-assembled membrane to its definitive morphology requirements while preserving the subject for operation. Today, widely used thorough sub-surface imaging techniques include ultrasonic atomic force microscopy (UAFM)^19–21^, X-ray^22^ and interferometry^23^. While such techniques provide a stack of 2-D profiles, they have their own limitations: UAFM and most interferometries have a limitation in subject thickness that could be inspected, interferometry cannot measure structures smaller than the wavelength of light used, and X-ray measurement resolution is larger than 100 nm^22^, not to mention the low-throughput of all these methodologies, ranging between tens of seconds to minutes. Therefore, a different approach must be considered to overcome the aforementioned intrinsic limitations. Sub-surface morphologies of SON and GON structures depend on the initial pattern, annealing condition and duration. It is noted, however, that under the same processing conditions, both sub-surface and surface morphologies are almost identically and consistently reproducible^1,24^. Thus, the necessity of the exhaustive inspection schemes for the purpose of sub-surface inspection may be questioned; instead, an accurate characterization of surface topography and the utilization of the correlation between the surface and the corresponding sub-surface could offer an alternative. Specifically, a sufficient database of surface and its corresponding sub-surface correlations and a robust methodology to interpolate a new surface image from the database would allow for a non-destructive and high-throughput (acquisition period less than few seconds) inspection scheme. This work undertakes quantifying and exploiting such correlation using principal component analysis (PCA). We further show that the PCA-based methodology can be employed not only for sub-surface analysis but also surface defect analysis. Indeed, PCA serves as a simple yet powerful tool to simplify and analyze the trend behind voluminous data, and has already been utilized in various biological domains: healthcare^25^, medicine^26^, and cell^27^ and virus^28^ analysis.

Taking advantage of such a competent tool, we wish to establish a novel methodology for: constructing a GON surface to sub-surface morphology database; employing the surface image to interpolate the sub-surface structure from the established database; and detecting and quantifying defects via surface projection on PCA plane. This work uses surface-fitted polynomial coefficients as the extracted features of the surface topography pattern, and will focus on GON structures. Annealed with the same initial pattern, GON and SON structures are known to temporally transform in a similar fashion^4,18^. Namely, annealing a 2-D lattice patterned initial structure yields either spherical voids or plate-shaped voids depending on the diameter, pitch, and depth of the initial pattern. Therefore, while this work’s classification scheme focuses on the temporal transformation of GON structures, the same methodology could be applied to SON structures with the same initial latticed pattern as well. Furthermore, we envisage the application of the proposed methodology’s approach for sub-surface analysis and quantitative defect analysis to any structures with a patterned surface profile.

## Methods

### GON fabrication

**Figure 1:**
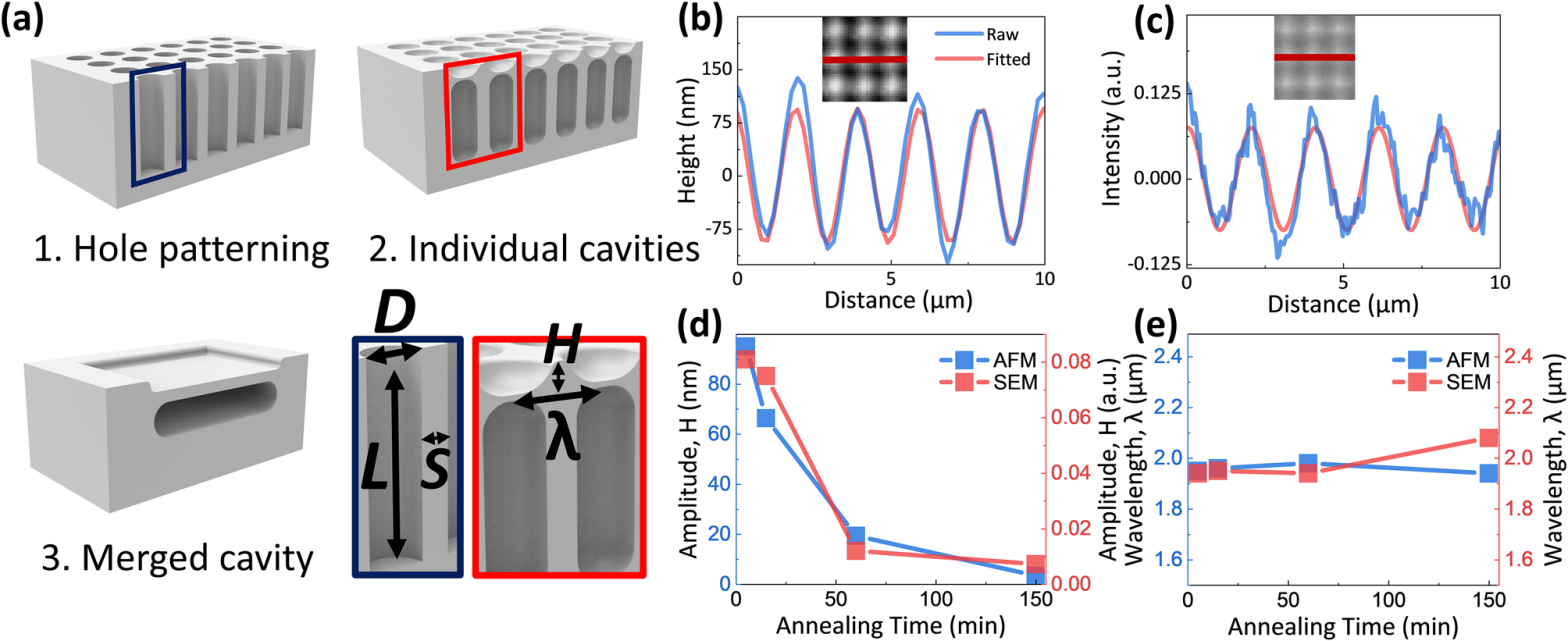
(**a**) The shape evolution process of the GON structure. As shape evolution proceeds, the height and spacing of the cavity changes, and steps and periodic structures are formed on the surface. Length (*L*) and diameter (*D*) are used to calculate the aspect ratio (*L/D*), and height (*H*) and period or wavelength ($\lambda$) to quantify the surface profile transformation over time. (**b**) AFM and (**c**) SEM images of periodic morphology formed on the surface of 5 min annealed GON structures and their sinusoidally fitted profile. The sinusoidal signal is zeroed by subtracting its temporal average. (**d**) Change in amplitude (*H* from **a**) of the sinusoidal fits over increasing annealing time. (**e**) Change in wavelength ($\lambda$ from **a**) of the sinusoidal fits over increasing annealing time.

As shown in Fig. 1a, hole patterns are fabricated on the surface of a prime-grade Czochralski (100) germanium (Ge) wafer. The diameter (*D*) and spacing (*S*) of the hole patterns in Fig. 1a are 1.2 ${\upmu }m$ and 0.8 ${\upmu }m$, respectively, with the aspect ratio (aspect ratio = *L*/*D*) of 12 where L denotes the hole depth. A diluted ammonia solution (${\hbox {NH}}_4$OH:${\hbox {H}}_2$O = 1:4 in volume) was used for 10 min to remove organic matters on the Ge surfaces. In addition, a diluted hydrogen bromide solution (HBr:$\hbox {H}_2$O=1:4 in volume) was used for 5 min to remove the native oxides on the Ge hole patterns^4^. The Ge hole patterns were annealed in a high vacuum furnace ($2 \times 10^{-6}$ Torr) at 890 $^{\circ }$C to accelerate the surface diffusion, and the initial hole patterns underwent shape evolution from individual cavities to a merged cavity as annealing proceeds, shown in Fig. 1a. The ramping rate of temperature was set to be 25 $^{\circ }$C/min. The cover of another cleaned Ge wafer was simply placed on the hole patterned sample to prevent the formation of thermally induced defects due to high temperature and high vacuum annealing^29^.

**Figure 2:**
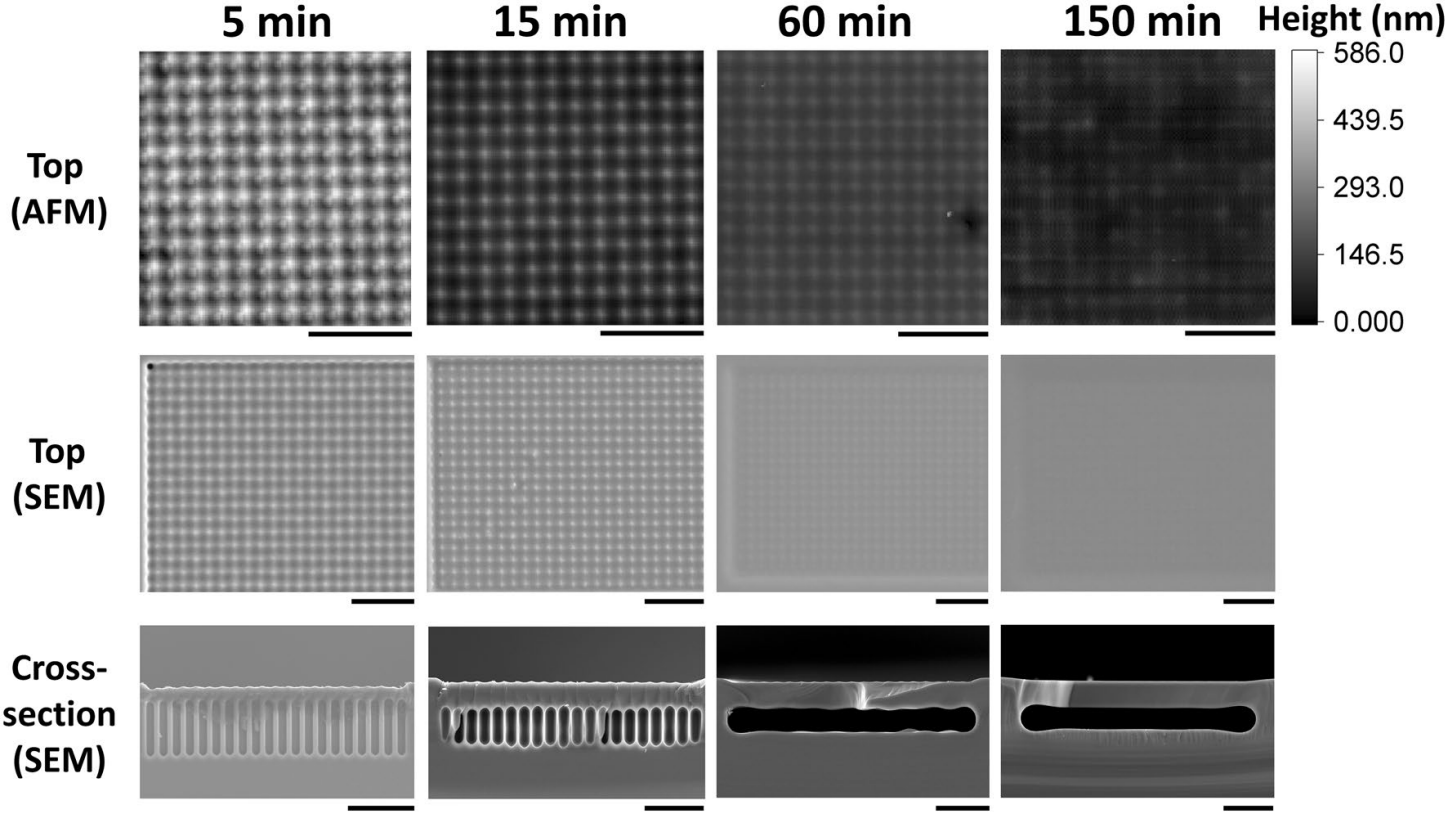
Surface AFM and SEM, cross-sectional SEM images of GON structures at each annealing time. As the annealing time increases, the shape evolution of the cavity occurs, resulting in the formation of individual vertical and merged horizontal cavities. Such change in cavity morphology could be noticed from the corresponding change in surface profiles. All scale bars are 10 ${\upmu }$m.

On the final GON structure surface, both lateral and vertical sinusoidal patterns have resulted from annealing the initial hole patterns, which are observed by both AFM topography and SEM image (Fig. 1b,c). As the annealing time increases, the height of the sinusoidally fitted periodic structure decreases (Fig. 1d) while the wavelength remains identical (Fig. 1e). The raw top and cross-sectional AFM/SEM images of the GON structure over increasing annealing time are compared in Fig. 2. As the annealing time increases, individual vertical cavities are formed due to surface closure, followed by their mergence into horizontal cavities as a result of surface diffusion. Such temporal development of sub-surface morphology is evidently correlated to the gradual decrease in surface topography variation (Fig. 1d), as shown in Fig. 2. The gradual decrease in surface topography variation could be quantified from AFM and SEM images. While AFM acquires an accurate physical change in depth, SEM expresses such change in height with change in image intensity. Taking advantage of the PCA-based database proposed in this work, the temporal transformation of AFM and SEM topographies is quantified, allowing a sub-surface morphology prediction based on AFM and SEM images. In addition, the correlation between AFM and SEM topographies are quantified.

### PCA database construction

**Figure 3:**
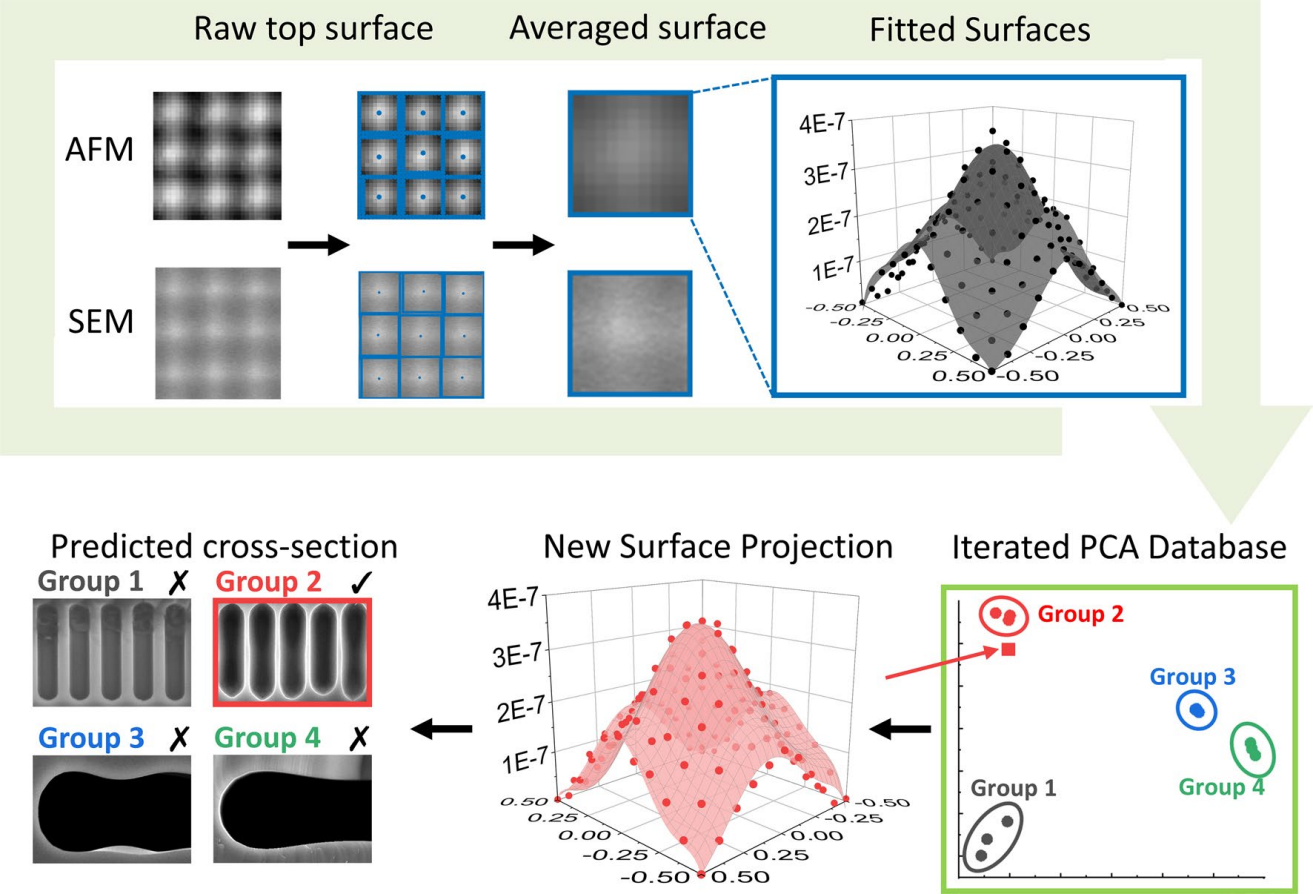
Overall pipeline of sub-surface analysis using PCA-based classification.

As illustrated in the overall schematic of Fig. 3, the local window surfaces are extracted by detecting the local maximum intensities of the surface images from a given structure. Then, 150 local windows are averaged for each structure, where each are fit to a polynomial surface function. For an intuitive analysis in search of a trend in the fitted surfaces, the dimensions of the fitted coefficients for each structure (3 structures for each of 4 annealing durations) are reduced via PCA to construct databases for each set of scanning electron microscopy (SEM) and atomic force microscopy (AFM) images. Then, a classification model assigns new SEM and AFM surface image to one of the clusters based on the PCA coefficients used to construct the database. Finally, each cluster’s sub-surface structure is determined based on an empirical decomposition. While this work only comprises four different clusters with varying annealing time for a single initial pattern, further accumulation of additional empirically decomposed structures and its corresponding surface profiles would increase the classifiable resolution of surface profile.

**Figure 4:**
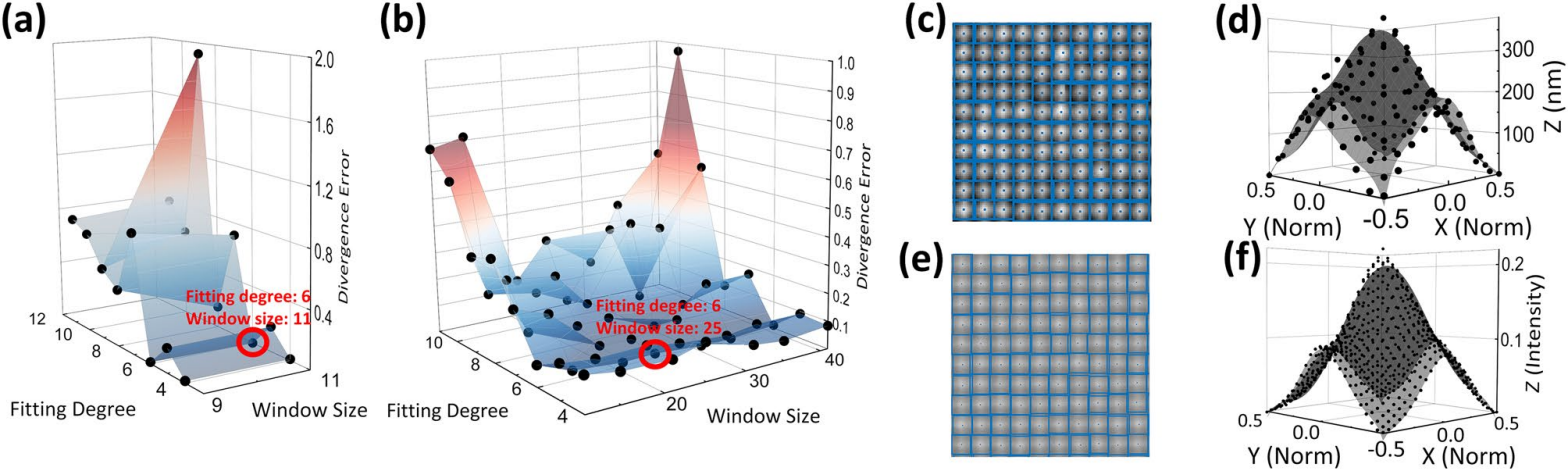
Illustration of the surface fitting procedure. (**a**, **b**) 3-D contour plots visualize the AFM (**a**) and SEM (**b**) surfaces’ PCA classification divergence error over two variables: surface fitting degree and window size. (**c**, **e**) Local windows for each individual inclined surfaces detected by finding the local maximums, respectively for AFM (**c**) and SEM (**e**). (**d**, **f**) Average surfaces fitted to the optimal conditions from (**a**), respectively for AFM (**d**) and SEM (**f**).

Figure 4 illustrates the data pre-processing process before PCA. From raw SEM and AFM images, local maximum points (maximum height for AFM image and maximum intensity for SEM image) are determined for each initial windows. The initial windows are calculated by dividing the image in manually determined ground truth window size, chosen as 41 by 41 and 11 by 11 pixels respectively for SEM and AFM. Then, each are refined by re-centering in reference to the local maximum position. Figure 4c,e shows the refined local windows. Subsequently, the averages of each structure’s windows are fit to a two-variable polynomial function with $- 0.5$ to 0.5 range for both variables. Then, the function coefficients are stacked for PCA. Specifically, 3 different GON structures for each 4 different annealing durations (5, 15, 60, and 150 min) are stacked. This work uses single variable decomposition (SVD) based PCA, a convenient tool implemented in MATLAB, to find the eigenvalue plane that expresses the maximum covariance of the input variables.

The PCA databases are constructed using the first two or three principal components obtained from conducting PCA on the stacked coefficients. For the scope of this work, use of only the first two was sufficient. The constructed PCA database quality depends on the degree of overfitting of the fitted surface, which could be controlled by adjusting the polynomial fitting degree and averaged surface’s resized window shape. Note that the averaged image is resized, not the ground truth window size mentioned above which is used when detecting local windows. Both parameters affect the degree of over-fitting of the averaged surface. If the fitted surface incorporates extraneous details, similar surfaces from the same annealing time structures are not closely clustered in the PCA dimension, as shown in Fig. S2. Therefore, these two variables are fine-tuned to yield the most accurate database by minimizing the divergence error shown in Fig. 4a. The divergence error is calculated using Eqs. (1, 2), which quantifies the divergence of data points from the same cluster.

1 $$\begin{aligned} div_a = \frac{\sum _{i,j\in A} ||(x,y)_i - (x,y)_j||}{\overline{||\overline{(x,y)_n} - \overline{(x,y)_m}||}_{(n,m)\in B}},\ div_b = \frac{\max ||(x,y)_i - (x,y)_j||_{(i,j)\in A} }{\min ||\overline{(x,y)_n} - \overline{(x,y)_m}||_{(n,m)\in B}} \end{aligned}$$

where the pairs *i*, *j* and *n*, *m* are all possible data point combinations for *x* (PC 1 axis) and *y* (PC 2 axis) within each annealing time cluster and for combinations for all annealing time cluster averages, respectively.

2 $$\begin{aligned} Div\ Error = \frac{div_a}{\max (div_b)} + \frac{div_a}{\max (div_b)} \end{aligned}$$

The first term of Eq. (1), $div_a$, represents the degree of divergence between each averages of the annealing time clusters. The second term, $div_b$ accounts for the degree of divergence between each structure projection of the same annealing time. In Eq. (2), each term is scaled to its respective maximum value, and the divergence error is determined as the sum of the normalized divergences.

**Figure 5:**
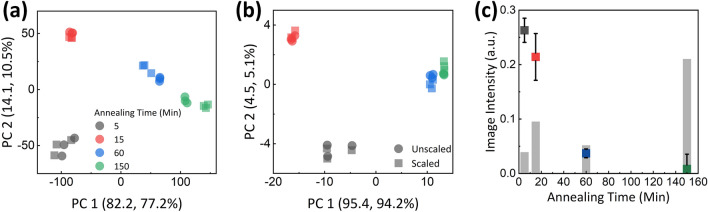
PCA analysis databases of AFM, SEM surfaces. (**a**, **b**) The coefficients of averaged local windows of AFM (**a**) and SEM (**b**). (**b**) images and the normalized mean deviation of true surface from fitted surface are reduced to two variables using PCA. The colors indicate annealing duration, and the shapes denote the scaled and unscaled mean deviation iterations of PCA. For the scaled iteration, mean deviation was scaled until it reached the largest significance among the PCA variables. (**c**) Colored boxes represent the maximum height of each annealing phase surface with their corresponding error bars indicating the maximum height’s standard deviation. Grey columns represent the normalized mean deviation for each annealing phase, which is used as the additional variable for PCA.

The parameters that yield the lowest divergence error were window size of 25 by 25 pixels and exponent degree of 6 for SEM image surfaces, and window size of 11 by 11 pixels and exponent degree of 6 for AFM surfaces. Fig. 4d,f show fitted surfaces of each 5 min annealed structure surface images from AFM (Fig. 4d) and SEM (Fig. 4f). In addition to the surface fitting coefficients, mean deviation of the fitted surface from the true surface also exhibited an annealing time-dependent trend, as shown in the grey columns of Fig. 5c. For structures up to annealing time of 60 min, the surface profiles exhibited a definite sinusoidal trend. On the other hand, the 150 min-annealed structure's surfaces were flattened out to a degree that sinusoidal trend was replaced by a randomized trend as the dominant profile. By nature, the fitted surface could not sufficiently account for the randomness of the flattened surface, and resulted in a large degree of mean deviation between the fitted and true surface. Using mean deviation as an additional parameter incorporated this trend when constructing the PCA database. Figure 5c illustrates such distinctively large mean deviation for 150 min annealed structures. For mean deviation calculation, each pixel’s deviation was normalized to its respective fitted surface height to account for the different scale in height for different annealing times. In summary, 6th order surface fitting yielded 28 surface fitting coefficients, and mean deviation was added as the 29th coefficient, which were conducted PCA as a set.

When projecting a new surface onto the PCA database for annealing time classification, even an identical structure of the database was projected differently under different surface image acquisition parameters. Indeed, both AFM and SEM not only incorporate experimental uncertainties but also discrepancy due to difference in acquisition parameters and environments, especially SEM. Unlike the experimental uncertainties, the acquisition discrepancies are accountable with robust post-processing of the acquired data. In terms of AFM, a contact-based measurement methodology where the absolute height is measured, nearly identical topographies are acquired for all measurements of the same sample. On the other hand, SEM images are additionally sensitive to image acquisition settings, therefore requiring a precise post-processing of the obtained images. The three main parameters that most influence an image condition are known to be focus, brightness, and contrast. In terms of focus, an out-of-focus images would need to be post-processed to fine-tune its focal plane. Since such topic is a separate on-going research topic, fine-tuning the focus is out of scope for this work and will only work with accurately focused images. To account for variation in brightness, each individual window is zeroed by subtracting the window by its minimum brightness. On the other hand, the remaining parameter contrast, which linearlly transforms the range of the image intensity, does not have a definite reference scale for normalization. Therefore, this work iteratively adjusts the individual window contrast until the projected new surface reached closest distance to a single annealing time cluster. Specifically, the contrast of each average windows is iteratively modified until the PCA plane Euclidian distance error towards a single annealing phase cluster centroid is minimized. The entire iterative process finds the modified minimum and maximum contrast threshold values that minimize the euclidean distance error to each cluster. For each iteration, the maximum gradient direction that minimizes the euclidean error is calculated. Then, the contrast thresholds are updated in accordance to the maximum gradient direction. The iteration ends when gradient is not found, or when the contrast reaches the threshold boundary. Such procedure is repeated for each annealing phase, and the new surface is predicted as the annealing period with the minimum iterated distance.

Figure 5a,b show the distribution of database surfaces using two variables, respectively for AFM and SEM. Circles denote the database constructed only with surface fitting coefficients, and squares the database with mean deviation added as an additional PCA variable. For the square databases, mean deviation was scaled until its absolute value reached the maximum among the 29 (28 surface fitting coefficients, 1 mean deviation) PCA coefficients’ absolute values. The percentage inside each axis label represents the proportion of covariance expression for the corresponding principal component variable, respectively for unscaled and scaled database. For both AFM and SEM databases, annealing time clusters of 60 and 150 min were most closely projected. Increasing the distance between these two clusters would increase the robustness of the classification of new surfaces with annealing time near the closely located clusters. Inclusion of the scaled mean deviation as the additional PCA variable precisely brought about such improvement.

For the scope of defect inspection, the database is established in a slightly different manner, with the sole difference in surfaces comprising the database. The specific defect morphologies of interest are handpicked to construct the database. Namely, one standard surface of 15 min annealed structures and two surfaces with defect of different morphologies are used. In addition, to account for rotational variation, each defect surfaces rotated by 90$^{\circ }$, 180$^{\circ }$, and 270$^{\circ }$ were also used as databases. Total of 9 surfaces were used in constructing the PCA database shown in Fig. 7d: two defect sets each comprised of a defect surface and its three rotational variants, and a single averaged surface. In doing so, specific defect structures of interest on a 15 min-annealed surface are quantified and detected in a selective manner.

### PCA database analysis

The PCA databases, both AFM and SEM, constructed with the aforementioned methodology bears minimal divergence error among the possible databases. Despite their qualitative resemblance, a quantitative analysis is additionally applied for further scrutiny. In doing so, the distance between all possible combinations of clusters: $_n C_r$, where n and r are both annealing durations, are calculated for each AFM and SEM database. Then, each set of distances are normalized to their respective maximum distance. Finally, the percentage difference of each corresponding normalized distances are averaged.

## Results

### New surface classification

**Figure 6:**
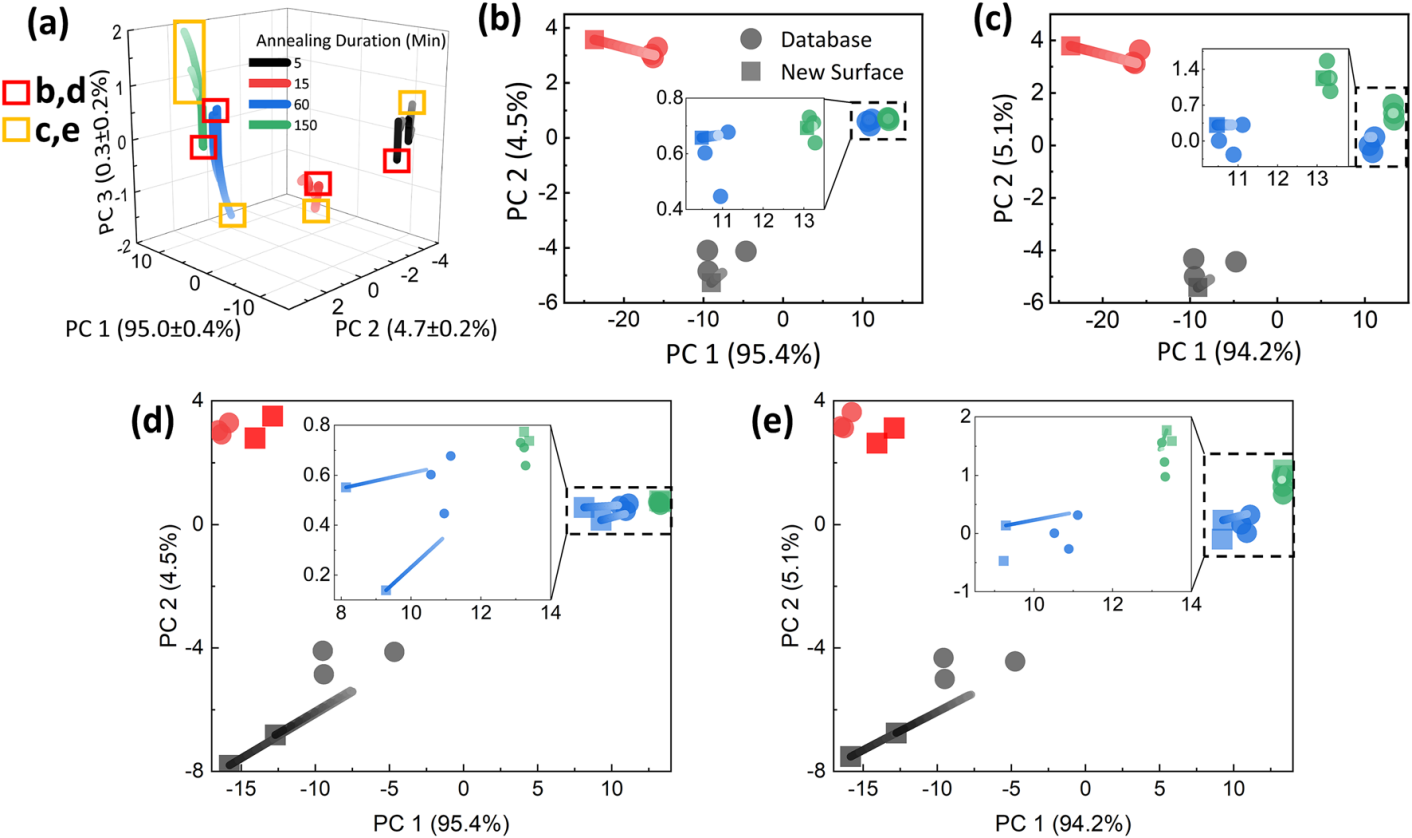
(**a**) 3-D plot of PCA database of SEM images over increasing surface fitting deviation scaling. Darker to brighter color denotes the increase in deviation scaling, from 1 to 26. The first three dominant principal components account for 100% of the PCA variance in average. (**b**, **c**) The projection and error minimization of synthetic experimental SEM images, respectively for unscaled (**b**) and scaled (**c**) database. Each figure demonstrate the iterative clustering on non-scaled and scaled databases, respectively. Circles represent the database, and squares the new surfaces. The lines illustrate the iterative classification for each new surfaces. Error is minimized in the direction of increasing brightness. (**d**, **e**) Experimental SEM images projected on PCA database, respectively for unscaled (**d**) and scaled (**e**) databases. For 5 min annealed devices, the experimental images were acquired with a different contrast setting.

Figure 6a shows the 3-D SEM database over increasing mean deviation scaling, where the direction of increasing brightness indicates increase in scaling. The separation of 60 and 150 min-annealed clusters is evidently exhibited when plotted in 3-dimensions. Figure 6b,c show the surface classification of synthetic new surfaces, respectively for unscaled and scaled databases. Specifically, the contrast of one image of each annealing duration was randomly adjusted, with their projections illustrated as squares in the figures. Then, each projection was iteratively classified, as explained in the methods section, to a single annealing cluster. All surfaces were accurately classified.

Figure 6d,e shows the surface classification of experimental new surfaces, respectively for unscaled and scaled databases. The surface SEM images were acquired on a separate structure which were annealed to the same iterations of duration. All structures were accurately classified to its respective ground truth cluster. Compared to the projections of synthetic surfaces, the projections of experimental surfaces exhibited larger deviation in general. However, the deviation was an acceptable degree of uncertainty, and did not hinder an accurate classification.

### Defect analysis

**Figure 7:**
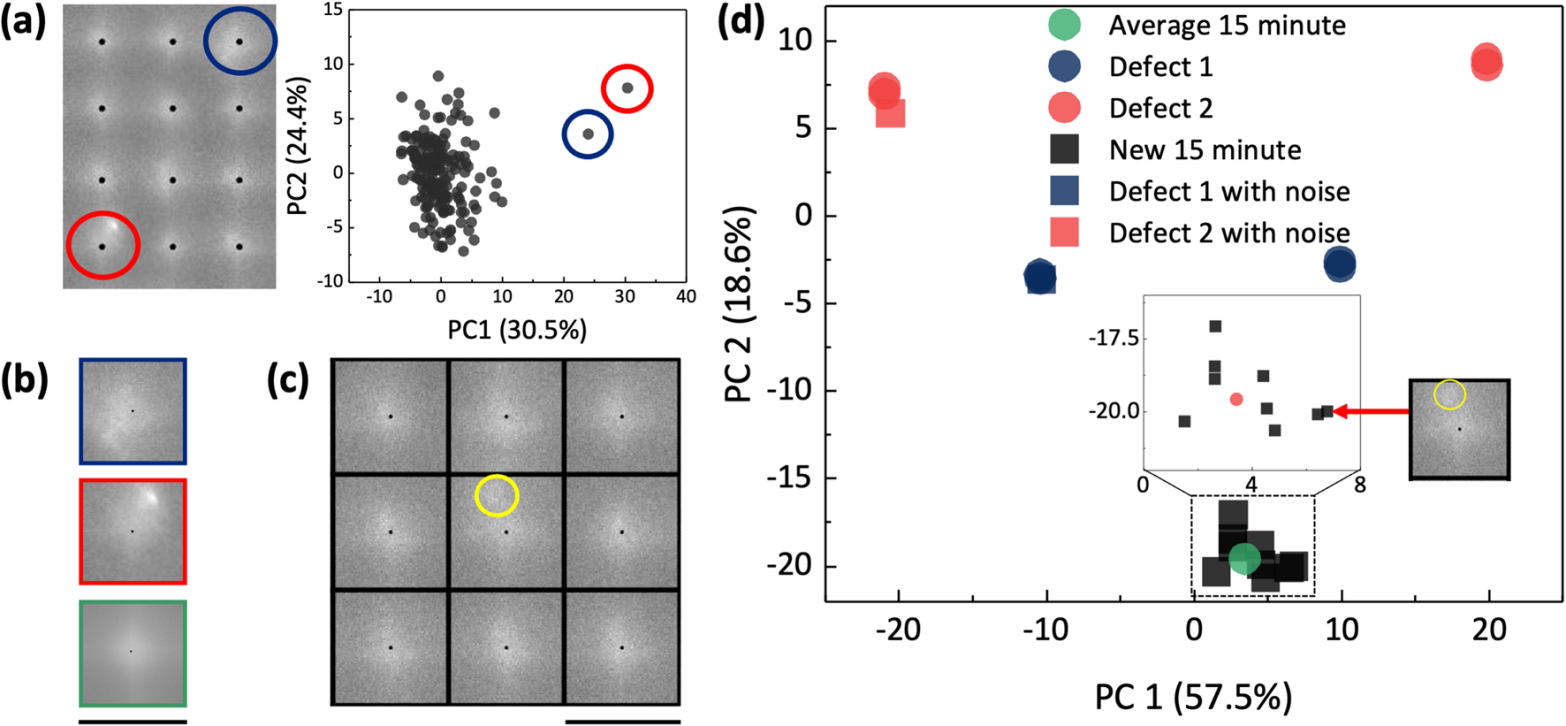
Illustration of defect detection scheme. (**a**) Naïve PCA on surface of a single 15 min-annealed structure detected two defects. The database is constructed with rotationally variated (90$^{\circ }$, 180$^{\circ }$, and 270$^{\circ }$) blue and red defects shown in (**b**). (**b**) Surface window of detected defects. Customized defect database was constructed using these two defects and one averaged surface of all windows of 15 min-annealed structure. (**c**) A segment of new 15-min structure for defect analysis on customized defect database. The yellow circle denotes presence of defect. (**d**) Defect analysis on customized database. While surfaces similar to defects 1 and 2 were classified as defects, all individual surfaces of new 15 min-annealed device were classified as regular 15 min-annealed surfaces. All scale bars are 10 $\upmu$m.

Figure 7 illustrates the performance of PCA-based defect detection methodology. Using a naive approach, all surfaces acquired from local max positions of a single structure’s surface were processed using PCA. One defect analysis-specific attribute to note is the fine-tuning process of detected local max positions. As shown in Fig. 7a, the surface image is rectified in a parallel manner, and the detected local max positions are line-arranged in reference to the nearby local max positions. Without the fine-tuning process for windows with defects, the local max positions are assigned to the location of the high-intensity defect, as marked by the yellow circles of Fig. 7c. Using these raw local max position without post-processing also successfully filtered out defects. However, a different approach was needed to further enlarge the scope. Namely, to effectively quantify and filter specific defect morphologies, local max positions that deviated greatly compared to other nearby local max positions were smoothened in reference to the nearby local max position. In doing so, local windows are sampled in a periodic lattice-like manner to incorporate the spatial morphology of defects with a definite reference point. As an experimental verification, 9 windows of a new 15 min-annealed structure shown in Fig. 7c along with two synthetic defect surfaces were projected onto the database. These structures were separately annealed under identical conditions as that of the structures used to construct the database. The synthetic defect surfaces were generated by adding noise with a maximal magnitude of 10% of the defect surface height. While the synthetic defects were classified to its corresponding defect clusters, all 9 windows of the new 15 min-annealed structure were classified closest to the average 15 min surface. Interestingly, the window with defect as noted with the yellow circle in Fig. 7c was also classified as a regular 15 min annealed surface. Such classification exhibits an effective filtering of the PCA database projection, with only hand-picked defect morphologies detected.

## Discussion

PCA served as a competent tool for reducing the dimensions and quantifying the trend of the voluminous surface fitting data. As described in the axis labels of Fig. 5a,b, the first two principal components accounted for 96.3% and 87.7% of the covariance for the AFM nonscaled and scaled databases, and 99.9% and 99.3% for the SEM nonscaled and scaled databases. Both AFM and SEM databases sufficiently expressed the variance between different annealing time surfaces using two variables. In particular, SEM databases achieved PCA accountability percentage over 99% for both scaled and unscaled databases. For future work, the addition of more experimental data for different annealing durations will further improve the classifiable annealing duration resolution of the database.

The PCA databases for AFM and SEM surface images exhibit high degree of resemblance, quantitatively proving the correlation between AFM and SEM surfaces. As shown in Fig. 5a,b, the 5 and 15 min annealed clusters exhibit nearly identical spatially distributed positions for both databases. Although the positions of 60 and 150 min annealed clusters are slightly modified, the overall trend in relation to the 5 and 15 min annealed clusters remain analogous. The separation trend of 60 and 150 min clusters with the inclusion of mean deviation as PCA variable is also shared by both. Therefore, the proposed methodology successfully quantifies the evident similarity between the AFM and SEM surface images in the projected PCA plane. When comparing the normalized euclidean distance between all possible cluster combinations, the average percentage difference between the AFM and SEM databases’ distances was acquired as 23.2%.

The proposed methodology resolves the key limitations of the previous sub-surface inspection methodologies: low throughput and limited measurable thickness and resolution. Interpolating from the SEM image PCA database only requires a single surface image, either AFM or SEM, reducing the acquisition period by a few orders with the use of the latter. Compared to exhaustive sub-surface inspection methodologies where acquisition durations are in the range of double to triple digit seconds^19–23^, acquisition of a single SEM image is instantaneous with the correct environmental setup, with less than a few seconds of data processing. In the future, utilization of the correlation between SEM images and optical microscope images would take one step further, once again increasing the process simplicity by only requiring a much easily accessible microscopic image. In regard of measurable thickness and resolution, an empirical destructive cross-sectional analysis resolves all limitations in measurable thickness and resolution. For example, taking a SEM image of the structure’s cross-section would allow an incomparably high-resolution sub-surface analysis. SEM image of a cross-section allows for lateral and depth resolution up to few nanometers^30^, which is also up to few orders higher in resolution compared to conventional thorough sub-surface analysis schemes. In addition, SEM image on any part of the cross-section could be taken, resolving any measurable depth limitations which were present with the prevalent wave-based non-destructive inspection schemes.

In terms of annealing duration classification uncertainty of GON structures, our methodology has shown 100% accuracy. Such perfect classification accuracy has been realized by averaging the local surfaces for a generalized specimen. When classifying individual local windows as shown in Fig. S1 and Table S2 of supplementary materials, the classification accuracy ranges between 80% and 100%. Instead of classifying GON structures based on single or few local windows, averaging 100+ windows resulted in 100% accuracy. Therefore, our proposed methodology accurately determines the annealing duration of GON structures based on surface topography only, from which sub-surface structures are empirically determined with cross-sectional analysis of sliced structures. Such cross-sectional analysis discovers a high-resolution sub-surface morphology, with uncertainty as low as single digit nm scale with SEM imaging. Compared to a traditional sub-surface inspection methodology such as infrared imaging which bears triple digit nm-scale uncertainty due to diffraction limit, our proposed work reduces the uncertainty by two orders.

The realms of application of the sub-surface analysis methodology for constructing and interpolating the database are not limited to GON structure surfaces, but to all surfaces with a periodic morphology such as Silicon-on-Nothing structures. PCA on fitted surfaces efficiently extracts the main contributors for deviation among the input data, therefore accurately quantifying the discrepancies between non-lineally transformed surfaces. The successful operation of PCA greatly depends on the quality of data pre-processing as proved by some adversary examples of Fig. S2. Specifically, the surface fitting parameters would need to be tailored to the specific problem to acquire maximal accuracy, one method being minimization of the PCA database divergence error as shown in Fig. 3a,b. Also, the window selection methodology requires fine-tuning to the surface-specific morphology. This work used GON structures which comprised of 2-D orthogonal initial DRIE patterns. Therefore, the annealed surface also exhibited a repetitive line arrangement. In the case of other patterns, a single window would need to encompass the entire repetitive pattern. If working with periodic structures with a more complex surface profile, the degree exponent of the fitted surface would need to be higher in general. In addition to the fitted surface profile, this work represented the deviation between true and fitted surface as a single averaged number. While such simple incorporation has sufficed for the scope of this work, a more detailed expression for mean deviation could be required for more complicated patterns. For example, a representation which encodes the spatial distribution of the deviation would provide a more rich information compared to a single mean deviation. A different approach for extracting features for PCA from more complicated non-linear topographies is a convolution-based autoencoder (AE) network. Although such deep learning-based approach adds an additional training step, complicated topographies are efficiently quantified as explained in the “Supplementary section” and as shown in Figs. S5 and S6.

In addition, the proposed novel methodology has not only shown competence in classification for sub-surface analysis but also for defect analysis. Even a naive approach of conducting PCA on all batch of individual windows successfully classified the outliers, as shown in Fig 7a. The subsequent approach broadened the scope, quantifying the defect morphologies and selectively detecting the defect morphologies of interest. Using this defect analysis scheme, the frequency of defects for any surfaces with a periodic morphology could be quantified. One of the main advantage of the proposed methodology is the selective filtering. When projecting new surfaces to the defect PCA database, only defects with similar morphologies to the selected defects are closely classified to the defect clusters. In Fig. 7c, the center local window comprises of a defect in the yellow circled region. However, when projected onto the PCA database, the corresponding surface was classified as a regular 15 min-annealed surface. Another advantage of the proposed methodology compared to other existent defect analysis schemes is the ease in algorithm tailoring. When constructing the defect database, one simply needs to select the types of defect morphology to discover and construct the database along with a regular surface. Compared to fully deep learning-based algorithms, our PCA-based defect detection process does not require exhaustive training procedure but provides the luxury of ease in tailoring the algorithm in respect to the desired defect morphology subject. Considering the manual labor resource required to annotate the training data for robust defect detection, our proposed work, which benefit from short acquisition period of few seconds, requires less temporal resources in at least three orders.

## Conclusion

This work proposes a novel methodology to quantify the GON surface patterns and analyze the sub-surface anatomy of GON structures based on SEM and AFM surface images. The proposed PCA based methodology not only successfully predicts the sub-surface structure based on a surface image of a new structure, but also quantitatively analyzes the distribution and degree of deviation of surface defects in a selective manner. The new approach resolves the low-throughput and inspectable depth and resolution limitations of previous non-destructive analysis methodologies for GON inspection. We envisage the introduced methodology to be used in diverse applications requiring device classification based on surface images and quantitative inspection of periodic surfaces.

## Supplementary Information

Supplementary Information.

## Data Availability

The datasets used and/or analysed during the current study available from the corresponding author on reasonable request.
