# Supplementary material for: PCA-based sub-surface structure and defect analysis for germanium-on-nothing using nanoscale surface topography
Source: Sci Rep. 2022 May 3;12:7205. doi: 10.1038/s41598-022-11185-w (PMC9065006; doi:10.1038/s41598-022-11185-w)
Supplement: Supplementary file 1 — Supplementary Information. [file 41598_2022_11185_MOESM1_ESM.pdf]

Supplementary information: PCA-based sub-surface structure and defect analysis for Germanium-on-Nothing using nanoscale surface topography

Jaewoo Jeong[1,2], Taeyeong Kim[1,2], Bong Jae Lee[1,2], and Jungchul Lee[1,2,*]

[1]Department of Mechanical Engineering, Korea Advanced Institute of Science and Technology, Daejeon, 34141, South Korea

[2]Center for Extreme Thermal Physics and Manufacturing, Korea Advanced Institute of Science and Technology, Daejeon, 34141, South Korea

[*]Corresponding author e-mail: jungchullee@kaist.ac.kr

1 Database analysis

Classification of each individual local windows incorporates inevitable uncertainties, including outliers. Therefore, instead of using single or a few local windows, the annealing duration of a given structure is determined on an averaged window from 150 local windows for an improved accuracy. To quantify the accuracy without average filtering, the individual windows are portrayed onto the PCA database constructed using the averaged window. Then, the percentages of each projected individual window closest to its correct corresponding annealing duration cluster are calculated, as shown in table S1. Figure S1 shows the individual windows portrayed onto the PCA database constructed with averaged windows. While individual window classification accuracy ranges between 80 and 100%, averaged classification shows full 100% classification accuracy as shown in the Results section of the main paper.

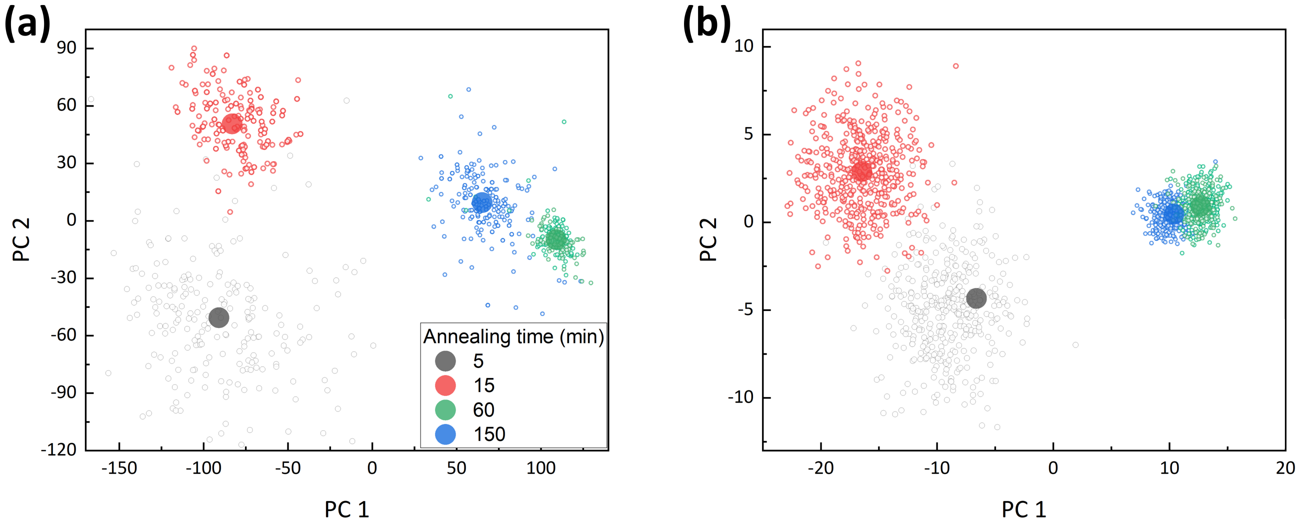

Fig. S 1: (a) AFM and (b) SEM databases (full circles) with projection of individual windows (empty circles).

Table S 1: Classification accuracy of individual windows to their corresponding annealing duration clusters of AFM, SEM databases.

Annealing duration (min)	5	15	60	150
AFM database (%)	93.9	100	91.7	97.2
SEM database (%)	83.8	99.1	85.1	87.8

The quality of the constructed PCA databases is dependent on the surface fitting parameters: fitting degree and window size. These parameters had to be heuristically fine-tuned to find their optimal values for efficient generalization of the local window surfaces. A smaller window size smoothens the surface as it resizes the local window surface, therefore filtering the unnecessary details. Especially for SEM images, the image resolution was considerably high, requiring a smoothening step for acceptable generalization of the surface. In terms of the fitting degree, although a higher fitting degree generally resulted in less deviation of the true surface from the fitted surface, such smaller degree had adverse effects on constructing an accurate database due to overfitting.

While the main manuscript showed the most optimal database, here we show additional databases with diverse quality. Figure S2 shows the PCA databases with the highest divergence error, respectively for AFM and SEM images. Unlike optimal PCA databases, at least one cluster is comprised of diverging datapoints. In addition, the percentage of variance represented by each first and second principal components were considerably lower to those of an optimal PCA database.

Figures S3 and S4 show more examples of above-mentioned optimal databases. Figure S3 shows additional databases for SEM surface images, and Fig. S4 shows additional databases for AFM surface images. For both SEM and AFM images, optimal databases were constructed using two fitting exponent degrees: 6th and 8th for SEM,

and 4th and 6th for AFM images. While the quality of the databases were similar for both exponent degrees for AFM images, the quality of SEM databases were slightly dependent on the fitting degree. As shown in the zoomed figures of Fig. S3, the 60 and 150 minute annealed clusters for SEM images were more separated with 6th degree exponent compared to 8th degree. Such is due to overfitting of the 8th exponent degree fitted surfaces.

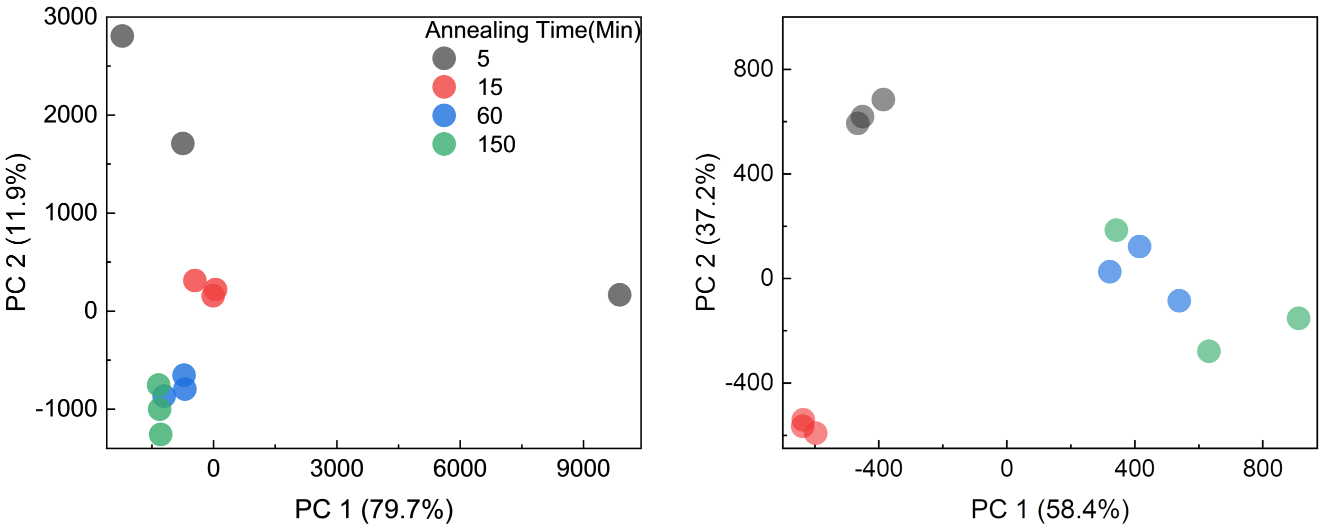

Fig. S 2: Examples of PCA databases constructed using the parameters with the high divergence errors shown in figure 4. Each AFM (left) and SEM (right) databases used 11,15 window size and 9,11 fitting degree, respectively. For similar surfaces, the high surface fitting degree resulted in large divergence on the PCA plane due to overfitting.

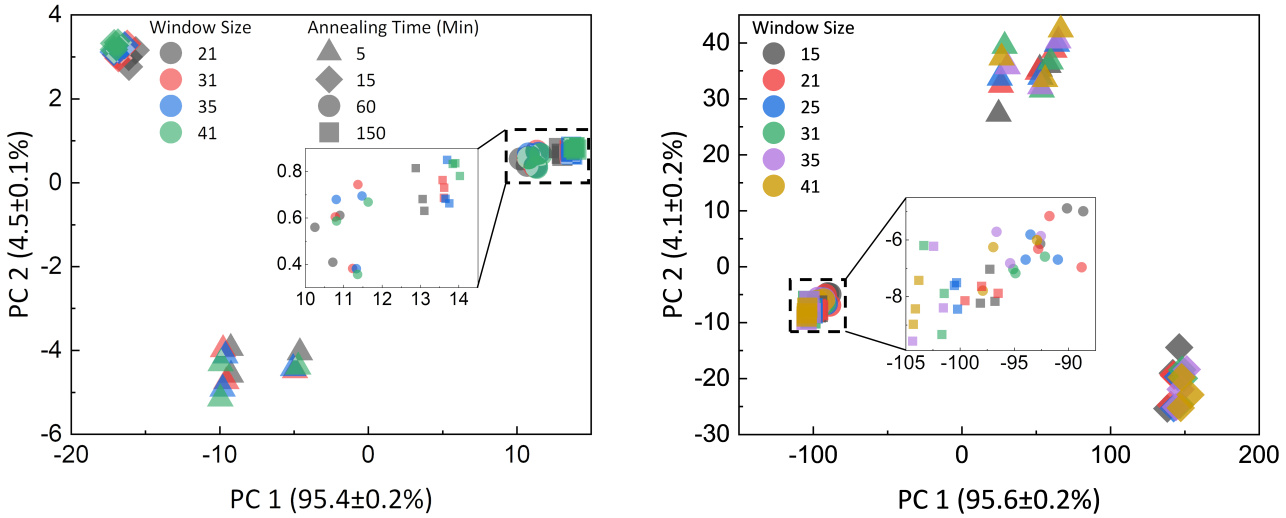

Fig. S 3: Example SEM PCA databases with lower divergence errors, lower than 0.01. 6th and 8th exponent degree surface fitting acquired low divergence error, each respectively represented by the left and right figure. The 6th degree database is slightly more robust compared to the 8th when considering the 60 minute and 150 minute clusters. Using 6th exponent degree fitting allowed for a more efficient generalization of the surfaces, resulting in a more definite separation of the 60 minute and 150 minute clusters.

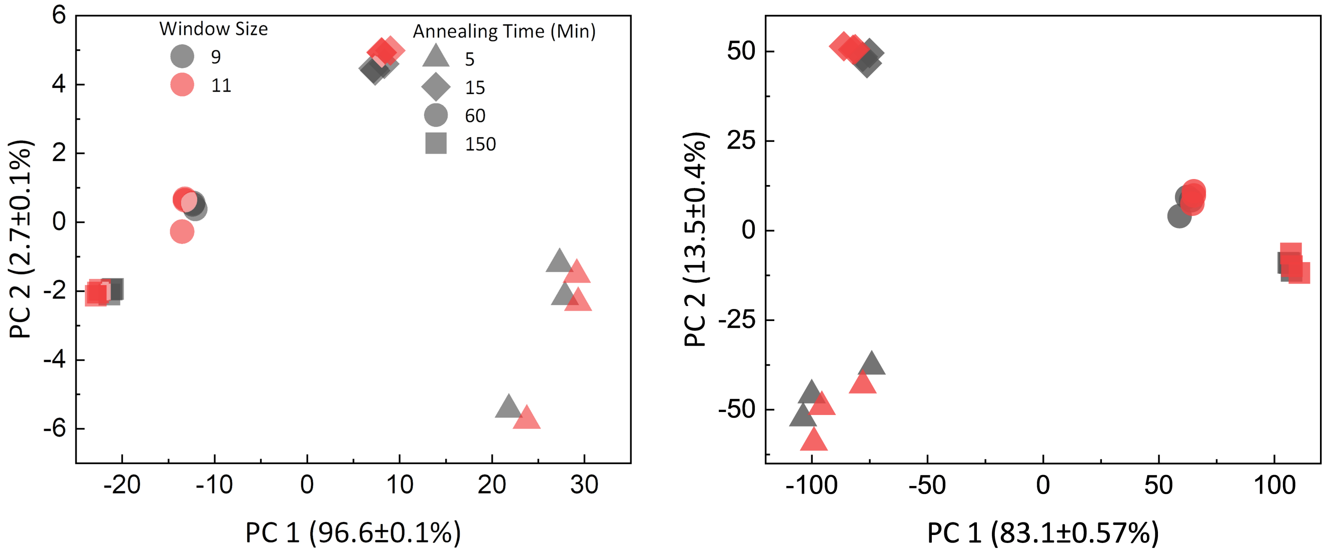

Fig. S 4: Example AFM PCA databases with lower divergence errors, lower than 0.01. 4th and 6th exponent degree surface fitting acquired low divergence error, each respectively represented by the left and right figure. Both databases were near equally robust, with similar distance between the annealing time clusters.

2 Autoencoder-based feature extraction for PCA

The surface windows extracted from the SEM, AFM topographies in the main work are simply hill-centered windows, with linearly decreasing height of the maximum slope height. In addition, the defects introduced in the main work also comprise of mostly simple morphologies, and therefore the surface fitting-based feature extraction methodology has sufficed. Namely, the main work has used the surface fitting coefficients as the extracted features of the window surfaces. However, to account for a more complicated morphology, a higher-order feature extraction methodology would be required. In this section, we introduce an Auto Encoder(AE)-based feature extraction method that could also be applied for PCA analysis of surface topographies with complicated morphologies. Figure S5 shows the overall schematic of AE-based classification for defects with complicated morphologies as shown in Fig. S6a. The autoencoder network is comprised of the encoder(blue) and decoder(green) network. The encoder network reduces the dimensions of the input defect image into a set of features. Then, the decoder network attempts to recreate the input defect image as close as possible based on the set of features. After training, the extracted features accurately express the input image with reduced dimensions. While this work has extracted 100 features for each 36 by 36 images, less number of features could be chosen to be extracted if desired. The extracted features are then conducted PCA, further reducing the extracted features into 2-D representations, allowing for an intuitive and efficient classification of defects with even complicated morphologies.

Figure S6a shows the defect images used for classification. The two variables for annealing conditions were annealing duration and annealing temperature, with other environmental conditions same as that described in the main section. The discrepancies between defects of different annealing duration are clearly distinguishable, in addition with subtle variations between defects of the same annealing condition. As shown in Fig. S6b, each clusters were located fairly separated from each other, showing the competence of the AE-based PCA classification for non-linear, complicated topography images.

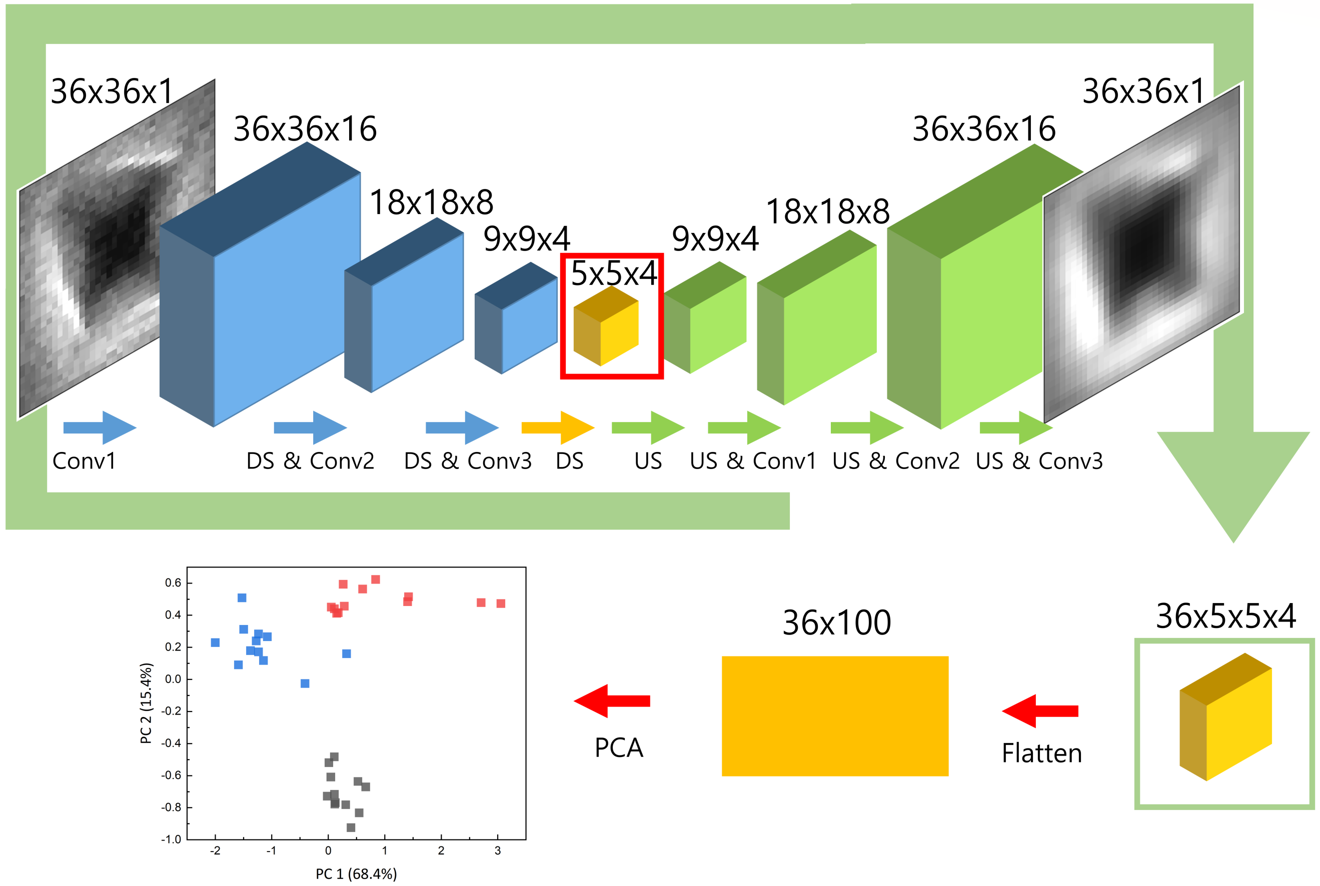

Fig. S 5: Illustrated schematic of the AE-based PCA classification methodology. The encoder(blue) and decoder(green) networks each comprises of three convolutional layers and three downsampling(DS) or upsampling(US) layers. The encoded information marked by the red box is retrieved as the extracted features of the defect image. The process is repeated 36 times, 12 images for each three annealing times. Finally, the features are flattened and conducted PCA to reduce the 100 dimensions into 2.

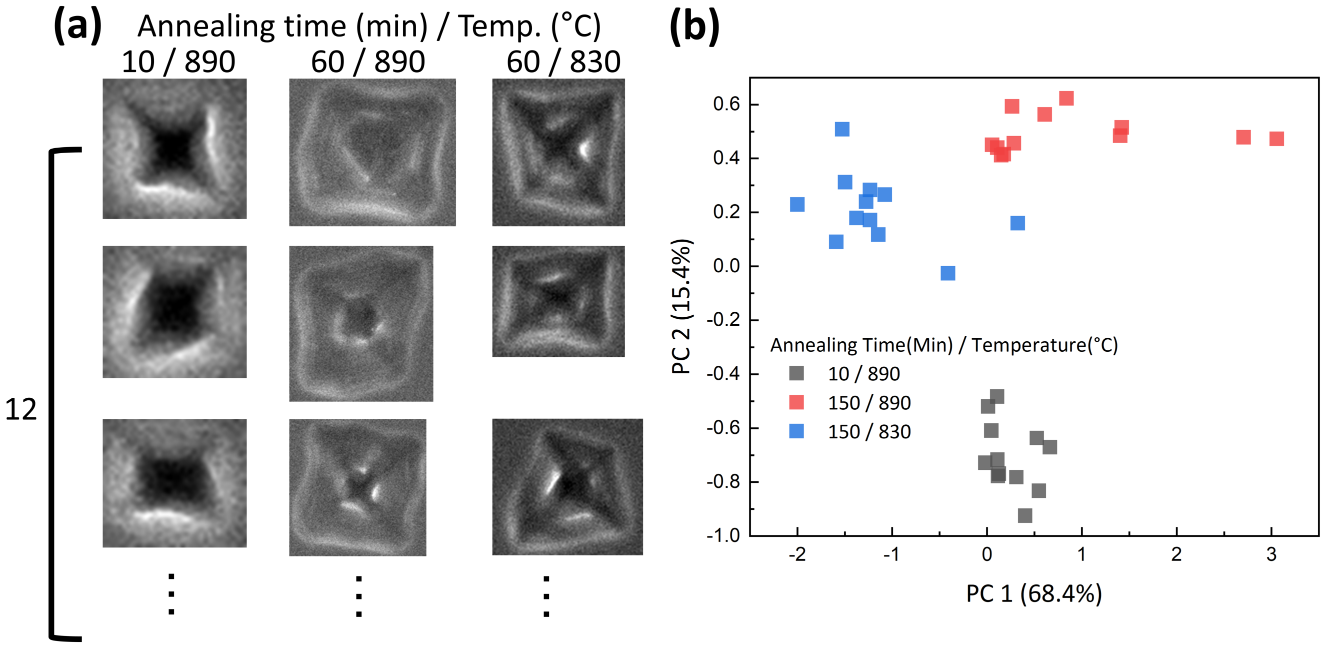

Fig. S 6: (a) Examples of defect images used for constructing the PCA database. Unlike the surface images used for the main work, the morphologies of the defect images used in constructing the AE-based PCA database are much more complicated. (b) The classified result of the AE-based PCA classification. Each cluster for the respective annealing conditions are separately clustered on the PCA plane.
